# A mini-review on the application of AI technology in the segmentation of maxillary sinus for dentistry

**DOI:** 10.3389/fsurg.2026.1793412

**Published:** 2026-05-20

**Authors:** Jiayi Chen

**Affiliations:** Department of Stomatology, Suzhou Wujiang District Hospital of Traditional Chinese Medicine, Suzhou, China

**Keywords:** AI, deep learning, dentistry, maxillary sinus floor, oral health

## Introduction

With the rapid development of modern technology, Artificial Intelligence (AI)—a product of the new technological revolution—has gradually assumed a vital role across various fields. The term “AI” refers to the ability of machines to perform tasks like humans. Machine Learning (ML), as a subdomain of AI, utilizes algorithms that can predict results from unseen data by learning intrinsic statistical patterns. Deep Learning (DL), as a subset ML, uses multi-layered calculations to learn and infer from data, particularly images (1). Over the past few years, DL has profoundly influenced several scientific disciplines. It has also been applied in state-of-the-art tasks like autonomous driving. For example, Tesla can perform autonomous driving on the expressway without supervision. DL also demonstrates vast potential in the biomedical domain. According to PubMed search results, biomedical studies generally focus on specialized medical scenarios, including the analysis of medical images such as computed tomography (CT) and pathological images (2). However, even though PubMed is the largest search engine in the medical domain at present, it cannot cover all medicine-related publications. For instance, medical publications are also disseminated through computer science conferences (3, 4). Prestigious annual conferences like Medical Image Computing and Computer Assisted Intervention (MICCAI) exert substantial influence on biomedical research. Compared with medicine, which has a millennia-long tradition, computer science is a relatively young discipline. Nevertheless, the combination of computer science and medicine will bring about revolutionary changes in the medical domain.

The paranasal sinuses consist of four parts: maxillary, ethmoid, frontal, and sphenoid cavities. They are filled with air, lined with mucosa, and communicate with the nasal cavity. The maxillary sinus was first reported by Leonardo da Vinci in 1489 and documented by the English anatomist Nathaniel Highmore in 1651. Therefore, it is also called the “Highmore” sinus (5). The maxillary sinus is a pyramid-shaped cavity located on the maxilla; it alleviates the weight of the maxilla. It is no longer acceptable to consider the maxillary sinus as two parts, containing an inferior part supporting the dentoalveolar process (the focus of dental practitioners) and a superior part related to the sinus ostium [the focus of Ear, Nose, and Throat (ENT) specialists]. Therefore, accurate recognize of the boundaries of the maxillary sinus on computed tomography/cone beam computed tomography (CT/CBCT) imaging is vital for both oral and maxillofacial surgery (OMFS) and ENT specialists. For example, maxillary sinus floor elevation is a classic technique in dental implantology, requiring dental practitioners to master the anatomy of the maxillary sinus floor. They should judge whether its floor is suitable to perform this surgery on CBCT images. The maxillary sinus puncture procedure is a classic surgery performed by ENT specialists to alleviate the inflammation of the maxillary sinus. It is important for specialists to evaluate the degree of inflammation of the maxillary sinus on CT images. However, reading and recognizing the anatomy and diseases of the maxillary sinus on CT images constitutes a challenge to young medical and dental practitioners. Recently, DL has been applied to the diagnosis and identification of the maxillary sinus on CT/CBCT imaging (6, 7). This state-of-the-art technology demonstrates the potential for assisting clinical practitioners in disease diagnosis.

## Sample size for DL

In general, the segmentation of regions of interest (ROIs) is an essential step of DL. However, manual delineation of ROI by physicians on CBCT/CT images is time-consuming and subjective, with the potential for bias between observers (8). Deep learning-based auto-segmentation depends on the availability of annotated datasets. It follows the general principle that the larger the datasets, the more accurate the results (9). However, high-quality medical images are scare, as they require the operator to master specialized medical knowledge and adhere to ethical principles. Therefore, it is vital to estimate the required training sample size before applying DL models to medical diagnosis. Insufficient sample size can lead to overfitting of training sets and underfitting of target tasks. If the sample size can be estimated based on the performance goals, it might help researchers design better models.

## Annotation of the sinuses

The air chamber of the maxillary sinus communicates with the nasal cavity via the ostiomeatal complex, which is made up of one or more ostia opening into the semilunar hiatus of the middle nasal meatus (10). In general, it is difficult to distinguish the maxillary sinus from the sinus ostium in medical imaging. Furthermore, the mucosa is thickened by inflammation of the maxillary sinus, which can lead to stenosis of the sinus opening. Due to these issues, it is a challenge for clinical physicians to annotate the boundaries of the maxillary sinus. Meanwhile, there are anatomic variations of the maxillary sinus—including maxillary septa, maxillary sinus dysplasia, and maxillary antroliths—which can affect the accuracy of annotation, resulting in the bias of segmentation when using the DL model.

## Clinical applicability

Integration of maxillary sinus segmentation algorithms into mainstream radiology software will offer time-saving and labor-saving advantages for physicians. Automated segmentation on radiography is particularly beneficial for junior physicians, graduates, and interns, as it can assist them in accurately identifying the maxillary sinus, thereby enhancing medical education. Recently, advanced algorithms with CBCT data have been applied to monitor the variation of maxillary sinus floor elevation with bone graft after several periods. Outstanding accuracy and rapid processing have been achieved using this algorithm (11). In the future, such algorithms are expected to be incorporated into workstations in the dental clinics. In addition, authors have applied the YOLOv11 framework to identify the posterior superior alveolar artery of the maxillary sinus, achieving an intersection over union (IoU) of 0.991 (95% CI: 0.948–0.974) (12).

## Discussion

I commend Busra et al. for their academic contribution entitled “Automatic segmentation of the maxillary sinus on cone beam computed tomographic images with a U-Net deep learning model” (13). This novel application has the potential to advance both otolaryngology and dentistry. However, from a dental perspective, there are several concerns that warrant consideration.

The maxillary sinus is not a completely hollow cavity within the maxilla; variations such as maxillary sinus septa and antroliths are frequently observed in CBCT images based on clinical experience. These anatomical variations are important factors in maxillary sinus floor elevation procedures for implant dentistry, as they may increase the risk of Schneiderian membrane perforation during surgery. Automatic segmentation of the maxillary sinus on CBCT images using deep learning algorithms could potentially reduce the incidence of membrane perforation. However, the authors did not address these factors in the inclusion or exclusion criteria of the study. Whether the U-Net framework can accurately predict different maxillary sinus morphologies with a high mean intersection over union (mIoU) or accuracy warrants further investigation in future studies.

Moreover, previous studies have demonstrated that training sample size has a significant impact on the performance of deep learning models for head-and-neck organ segmentation (14). However, the optimal sample size for deep learning models remains controversial. The U-Net framework, first proposed in 2015, is well known for its relatively low requirement for training samples in medical image segmentation tasks. In the present study, 100 axial CBCT images were analyzed, and the U-Net framework achieved an IoU value of 0.9275 and an F1 score of 0.9784. However, variations in maxillary sinus morphology across different planes, as well as the relatively small training sample size, should be further investigated in future studies.

In summary, this study is well designed and presents a novel approach. The integration of deep learning into maxillary sinus diagnosis is promising and may assist otolaryngologists and oral and maxillofacial surgeons in achieving more accurate diagnoses. Nevertheless, the limitations of the current model still require further investigation. With regard to the limitations, the authors noted that incorporating data from multiple centers could enhance the generalizability of the model. I further suggest that CBCT images collected from different countries and regions be included to ensure the model's generalization performance. Establishing a publicly accessible CBCT database may also promote global research in this field.

## Clinical significance

Reliable AI-assisted maxillary sinus segmentation could support clinical decision-making in implant dentistry and otolaryngology, reduce operator dependency, and improve the safety and precision of sinus-related surgical procedures.
